# Experience sampling method studies in physical activity research: the relevance of causal reasoning

**DOI:** 10.1186/s12966-025-01723-w

**Published:** 2025-03-05

**Authors:** Louise Poppe, Annick L. De Paepe, Benedicte Deforche, Delfien Van Dyck, Tom Loeys, Jelle Van Cauwenberg

**Affiliations:** 1 Department of Public Health and Primary Care, Ghent University, Ghent, Belgium; 2 Department of Experimental Clinical and Health Psychology, Ghent University, Ghent, Belgium; 3 Department of Movement and Sports Sciences, Ghent University, Ghent, Belgium; 4 Department of Data-Analysis, Ghent University, Ghent, Belgium; 5 School of Public Health, Université Libre de Bruxelles, Brussels, Belgium

**Keywords:** Experience sampling method, Ecological momentary assessment, Causality, Directed acyclic graph, Identification, Within-person encouragement design

## Abstract

**Background:**

The experience sampling method (ESM), also known as ecological momentary assessment, is gaining popularity in physical activity research. This method involves assessing participants’ behaviors and experiences repeatedly over time. One key advantage of ESM is its ability to temporally separate the dependent and independent variable of interest, reducing the risk of reverse causality. However, temporal separation alone is insufficient for establishing causality. This methodology paper illustrates the importance of the identification phase in drawing causal conclusions from ESM data. In the identification phase the causal effect of interest (or estimand) is specified and the assumptions under which a statistical association can be considered as causal are visualized using causal directed acyclic graphs (DAGs).

**Methods:**

We demonstrate how to define a causal estimand and construct a DAG for a specific ESM research question. The example focuses on the causal effect of physical activity performed in real-life on subsequent executive functioning among older adults. The DAG development process combines literature review and expert consultations to identify time-varying and time-invariant confounders.

**Results:**

The developed DAG shows multiple open backdoor paths causing confounding bias, even with temporal separation of the exposure (physical activity) and outcome (executive functioning). Two approaches to address this confounding bias are illustrated: (1) physical control using the within-person encouragement design, where participants receive randomized prompts to perform physical activity in their natural environment, and (2) analytic control, involving assessing all confounding variables and adjusting for these variables in the analysis phase.

**Conclusions:**

Implementing the identification phase enables ESM researchers to make more informed decisions, thereby enhancing the validity of causal inferences in studies aimed at answering causal questions.

**Supplementary Information:**

The online version contains supplementary material available at 10.1186/s12966-025-01723-w.

## Introduction

Studies using the experience sampling method (ESM), also termed ecological momentary assessment studies or diary studies, are increasingly popular to examine behaviors and experiences varying within individuals. In this type of studies, people’s actions, thoughts, emotions, and/or experiences are intensively assessed in real time and within people’s natural environments, contributing to the study’s ecological validity [1]. ESM studies typically provide detailed data at the individual level, which can be used to answer various research questions.

Research questions can be categorized into three broad groups [2]. First, there are descriptive research questions. In the context of ESM data, one could be interested in visualizing how physical complaints fluctuate over days among older adults [3]. The second type of research questions focuses on prediction. For example, Reiter and Schoedel predicted compliance to ESM prompts based on more than 400 variables [4]. Finally, there are causal research questions or questions focusing on whether, and if so to what extent, one variable affects another variable. For example, one could aim to investigate whether physical complaints reported in the morning affect levels of physical activity (PA) during the day among people with type 2 diabetes [5]. In this paper we focus on such causal questions.

The ultimate aim of many ESM studies is to investigate the relationship between potential intervention targets and their associated outcomes. Examples include the relationship between PA (as intervention target) and cognitive performance (as outcome) [6], the relationship between PA and affect [7], and the links between determinants proposed by health behavior theories and PA [7, 8]. Establishing the causality of these relationships is crucial to ensure their relevance for intervention design. However, in line with the prevailing notion that manipulation is needed to imply causation, researchers conducting ESM studies tend to refrain from using causal language in their study aims and results [9]. Consequently, predictive rather than causal analyses are used to answer the research question, but causal conclusions inevitably surface when translating the findings into policy and practice recommendations [10, 11]. This mismatch results in ambiguity, bias, and misinterpretation of research results [11, 12], also in ESM studies [9]. For instance, temporal within-subject relationships identified through predictive analyses (e.g., between self-efficacy and PA [13], or between PA and cognitive functioning [6]) are often mistakenly interpreted as evidence that the first variable is a meaningful intervention target for influencing the second.

Advances within the fields of Epidemiology and Computer Sciences have resulted in a formal framework providing insight under which assumptions causal conclusions can be drawn from observational data [14]. A key feature of causal analysis is the identification phase, which occurs well before considering any statistical analyses (estimation phase) [15]. In this identification phase, the causal effect of interest (or *causal estimand*) is specified and the assumptions under which a statistical association can be considered as causal are identified. *Causal directed acyclic graphs (DAGs)*, also known as causal diagrams, visualize these assumptions by displaying the assumed causal structure between the exposure (i.e., the independent variable) and outcome (i.e., the dependent variable) of interest and related variables [16]. As a result, DAGs provide information on which confounding pathways should be eliminated before a statistical association between the exposure and the outcome could be interpreted as a causal effect.

This paper aims to demonstrate the importance of incorporating an identification phase when developing ESM studies with causal research questions. We will focus on how the identification phase can help researchers make design-related and analytic decisions to reduce the impact of confounding bias. In the Methods section we describe how to define an estimand and demonstrate the relevance of creating a DAG. In the Results section we discuss two approaches to control for the identified confounding pathways. Throughout the paper, we deliberately refrain from offering guidance on the estimation phase. Our rationale for doing so is two-fold. First, by focusing on the identification phase, we hope to convey that it merits equal, if not greater, consideration compared to the estimation phase and should not be perceived as a subsidiary component of data analysis. While the estimation phase often receives significant attention, the identification phase is essential to ensure that the chosen estimation method (e.g., logistic regression with covariate adjustment) aligns with the causal question at hand [17]. Second, by not describing specific estimation techniques, we aim to highlight the generality of the identification phase, allowing researchers to select estimation techniques that best suit their specific data and assumptions.

To illustrate the identification phase, we use the following research question as use case: “What is the causal effect of PA performed in the natural environment on subsequent executive functioning (EF) among community-dwelling older adults?”. Cognitive performance is known to fluctuate daily in older adults [18], and lab-based studies suggest that PA can have acute effects on their EF [19]. However, the impact of PA performed in naturalistic settings on subsequent EF remains unclear.

## Methods

### Defining the estimand

A pivotal, but often neglected step in answering a research question is formulating the theoretical estimand or the target of inquiry without direct reference to the data at hand and the statistical analysis technique one aims to use (e.g., logistic regression) [17]. A theoretical causal estimand consist of two components, a contrast of potential outcomes and the target population [17]. Hence, by outlining this estimand we provide information on the outcome of interest, the target population and the levels of the exposure to be compared. For example, “the difference in the proportion of individuals being diagnosed with dementia (outcome of interest) among German adults aged 60–80 (target population) if they reached the WHO guidelines on PA at the age of 50 versus if these same adults did not reach the WHO guidelines on PA at the age of 50 (levels of the exposure to be compared)” is a theoretical causal estimand. Ideally, the exposure and outcome are clearly situated in time. Within the context of ESM, the exposure and outcome of interest are often time-varying within individuals [9]. In this case, we contrast the outcome for situations for which different levels of the exposure were observed within individuals.

#### Illustrative example for defining the estimand

Our research question at the outset needs to be further specified and transformed into a theoretical causal estimand. Our reason to focus on EF is threefold. First, EF has shown to fluctuate over time and contexts among older adults [18]. Second, acute PA enhances activity in the prefrontal cortex, a key structure for EF [20]. Third, lab-based studies examining the acute effect of PA on cognitive performance have mainly focused on EF [21]. EF is, however, a multidimensional construct, encompassing several high-level cognitive abilities, amongst which planning, decision-making, working memory (WM), responding to feedback, inhibition and flexibility [22]. Because WM deficits are the most often reported cognitive complaint among older adults [23], it was decided to focus on the subdomain WM. Hence, the outcome of interest is WM performance.

Similar to EF, PA is a multidimensional construct encompassing intensity, duration, frequency and type of activities performed [24]. Lab studies examining the effect of PA on cognition have typically focused on the effect of different PA intensities and durations on cognitive performance [21]. Among healthy older adults, immediate cognitive enhancements can be expected when PA is performed at a moderate intensity or higher [19]. Lab studies conducted among different age groups have found that PA performed for a period of ≥ 11 min is more likely to result in changes in cognitive performance than PA performed for a period of < 11 min [25]. Furthermore, lab-based studies have shown that stronger cognitive enhancements can be expected when the interval between the bout of PA and the cognitive assessment is less than 20 min [25]. Hence, the levels of the exposure are defined as performing 0 versus ≥ 11 min of PA at a moderate to high intensity in the natural environment in the 25 min (11 + 14 min) interval before the assessment of EF. An interval of 14 rather than 20 min was chosen to allow for sufficient time to complete the cognitive assessments.

Finally, the population of interest is defined as Flemish community-dwelling adults aged 65 or above who (1) show no indications of mild cognitive impairment, (2) are able to be physically active at a moderate intensity or higher, and (3) are able to complete cognitive assessments using a smartphone. Taken together, the theoretical causal estimand is “the difference in WM performance assessed maximum 14 min after performing 0 or ≥ 11 min of PA at a moderate to high intensity in the natural environment among Flemish community-dwelling older adults”.

After defining the estimand, one can decide on the assessment of the exposure and outcome. Here, the exposure as well as the outcome will be assessed in participants’ natural environment. WM performance will be measured using cognitive tasks provided on a smartphone. Ideally, more than one task is used to assess a cognitive construct [26]. Furthermore, in the context of ESM, these tasks need to reliably capture within-person changes [27]. Memory updating tasks asking participants to memorize and update (by addition or subtraction) numbers and spatial 3-back tasks are both valid tasks for assessing WM performance in older adults [26]. Furthermore, both tasks have shown to capture day-to-day variability in WM performance reliably among older adults [26, 28]. Considering participant burden, WM performance will be assessed using a memory updating task and a spatial 3-back task provided once per day. The measure of performance for both tasks is the percentage of correct responses [28]. The exposure of interest will be measured using accelerometry. Both PA intensity and duration will be assessed using a wrist-worn accelerometer as wrist-worn sensors are considered most suitable for long-term use among older adults [29]. Wrist accelerometers provide good validity and reliability for measuring PA duration at different intensities in older adults [30].

### The relevance of creating a DAG

By developing a DAG we create a better understanding of how the exposure and the outcome are causally linked with other variables. Furthermore, DAGs inform us which variables we should (not) adjust for to obtain an unbiased estimate of the causal effect of the exposure on the outcome. With adjusting for a variable we refer to statistically controlling for a third variable or a set of third variables in order to eliminate non-causal association between the exposure and the outcome. This can be done using different methods, amongst which outcome regression, propensity score analyses (e.g., inverse probability weighting) and stratification [16].

**Fig. 1:**
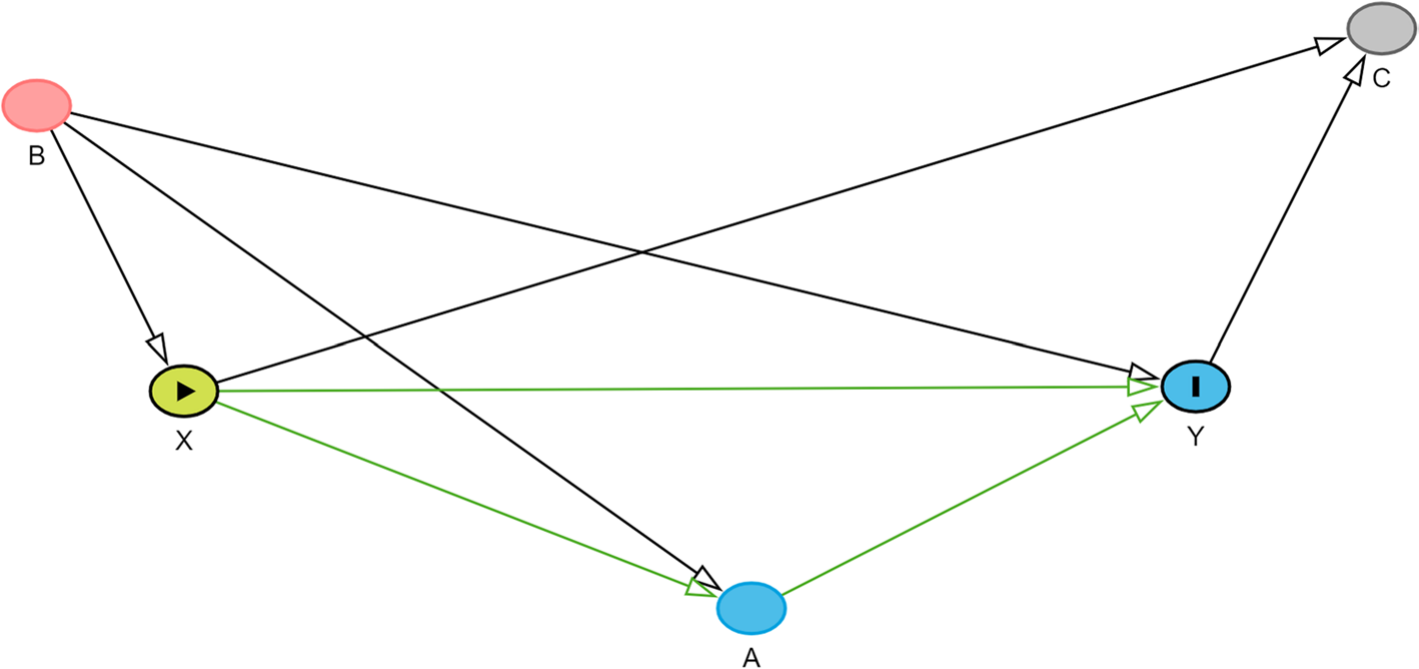
DAG with X as exposure and Y as outcome. Notes. Green arrows represent causal paths; A red node indicates a common cause of the exposure and the outcome; DAG created using Dagitty [32]

Figure 1 shows a simple DAG, in which variable X is the exposure and Y is the outcome. We will use this DAG to provide a brief overview of DAG terminology and rules. Variables are indicated using *nodes* and causally linked using *edges (arrows)*. A first key feature of a DAG is that all edges are *directed*. Hence, for each relation between two variables, the direction of the causal effect is explicitly indicated. A second key feature is that DAGs are *acyclic*, indicating that variables cannot cause themselves at the same timepoint [16]. *Directed or causal paths* are paths in which all arrows point in the same direction [14]. Here, the paths “X → Y” and “X → A → Y” are both directed paths. They represent a causal association between X and Y. *Backdoor paths* between the exposure and the outcome are paths with an arrow pointing into the exposure [31]. For example, “X ← B → Y” is a backdoor path. Backdoor paths linking X and Y represent non-causal association between both variables. Paths might contain a *collider* or a node having two edges pointing directly towards it. Here, C is a collider.

Paths can be *blocked* or *unblocked*. Paths without colliders are unblocked and therefore create statistical association between two variables. Here, the backdoor path “X ← B → Y” is open, representing *confounding bias*. B is a common cause of the exposure and the outcome and is thus a confounding variable. Adjusting for a non-collider variable (such as a confounding variable), blocks the path, eliminating the statistical association along the path. In our example, adjusting for B (e.g., by adding B as a covariate in outcome regression) would eliminate the confounding bias. Paths including one or more colliders are blocked and therefore do not produce statistical association between two variables. In our example, the path “X → C ← Y” does not produce statistical association between X and Y. However, adjusting for a collider opens the path, producing statistical (non-causal) association along the path. In our example, adjusting for C would produce a statistical (non-causal) association between X and Y (i.e., collider bias). In order to understand sources of confounding for a specific causal question, it is crucial to add relevant common causes of the exposure and the outcome to the DAG [33]. The selection of these common causes should be based on domain knowledge, which can be derived from the literature or by consulting domain experts [34, 35].

#### Illustrative example for developing a DAG

**Fig. 2:**
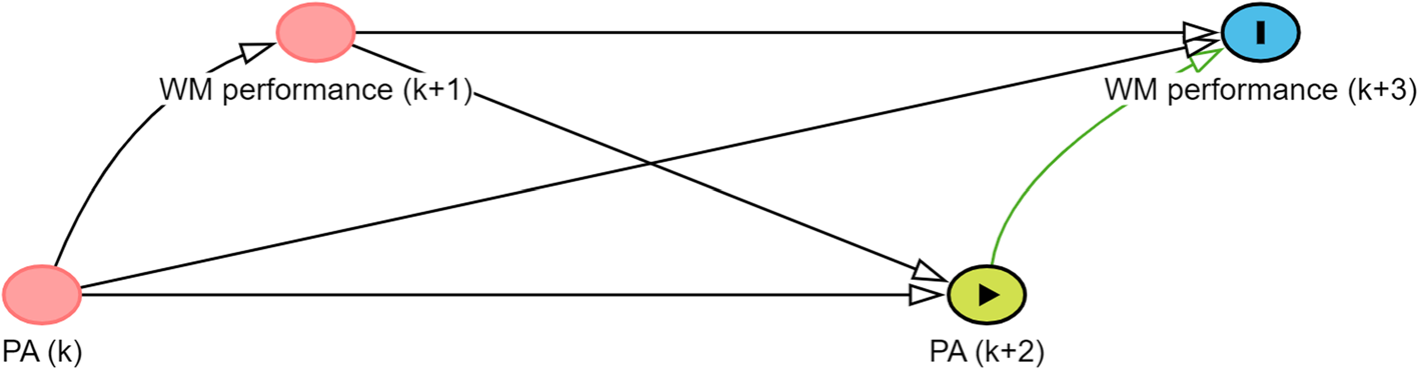
DAG depicting the time-varying exposure and outcome. Notes. k refers to the timepoint of assessment; DAG created using Dagitty [32]

We aim to examine the difference in WM performance assessed maximally 14 min after participants spent ≥ 11 min of PA at a moderate to high intensity versus 0 min of PA at a moderate to high intensity. Hence, an arrow from PA to WM performance is drawn [36]. Furthermore, because PA and WM performance are time-varying variables, a DAG depicting repeated assessment over time is created. Figure 2 shows the DAG depicting our time-varying exposure and outcome over a period of two days. The assessments of PA (k) and WM performance (k + 1) take place on the day preceding the day on which PA (k + 2) and WM performance (k + 3) are assessed. In reality, both PA and WM performance will be assessed over a longer period. However, for simplicity, we draw the DAG depicting two days of the study. Because not drawing an arrow is a stronger assumption than adding an arrow, arrows are also drawn from WM performance (k + 1) to PA (k + 2) and from PA (k) to WM performance (k + 3). It should be noted that we are not interested in the cumulative effect of the exposure over time. Instead, we focus on the immediate impact of PA performed in a natural environment on WM performance. This is indicated by the green arrow connecting PA (k + 2) and WM performance (k + 3). As we will repeatedly examine the exposure and outcome within individuals, a green arrow could also be drawn between PA(k) and WM performance (k + 1). However, for illustrative purposes, we will visualize relevant variables to identify the causal effect of PA on WM performance on one day. The next step is to add common causes of the exposure and the outcome based on domain knowledge. In within-subject designs, two types of confounders can be distinguished, namely time-invariant confounders (e.g., sex) and time-varying confounders (e.g., fatigue).

Input from the literature as well as from domain experts was gathered. The evidence synthesis method of Ferguson and colleagues was applied to select common causes (i.e., confounding variables) based on empirical studies identified by a literature search [34]. Because of the limited number of ESM studies examining the effect of PA performed in a naturalistic setting on WM performance, it was decided to focus on the more general domain of EF. Three studies examining the effect of PA performed in (older) adults’ naturalistic environment on EF at day-level were identified [6, 37, 38]. Supplementary File 1 describes the evidence synthesis in detail. The logbook documenting the specific steps taken during this process is provided in Supplementary File 2. The common causes obtained via the literature search were all time-invariant variables (i.e., age, sex, socio-economic status, body mass index, physical functioning, and baseline PA levels). Consequently, the potential impact of time-varying confounders was not taken into account in the selected studies.

**Fig. 3:**
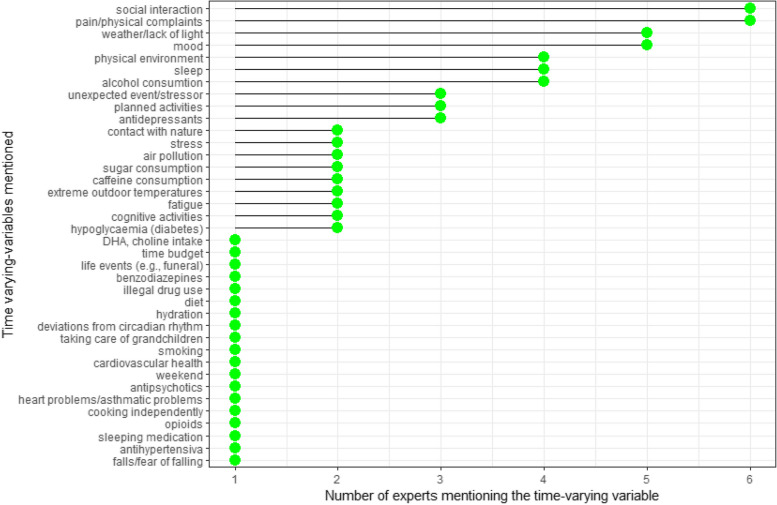
Time-varying variables mentioned by the experts

Six domain experts were consulted to identify relevant time-varying confounders. The experts all had specific expertise in PA and/or cognitive performance among (older) adults and had varying academics backgrounds (i.e., Psychology, Movement and Sports Sciences, Medicine, Epidemiology, and Statistics). Each expert was individually interviewed via online (5) or face-to-face (1) meetings. For experts with no or limited experience with DAGs, the meeting started with a short introduction on DAG terminology and their use (see Supplementary File 3). Because we focused on EF in the literature review, we also used EF as outcome in the discussions with the experts. All experts were shown a basic DAG visualizing the exposure and outcome over time via the online software ‘Dagitty.net’ [32] and asked which variables could influence both the exposure and outcome at day-level. Variables identified by the experts were immediately added during the meeting. As a result, six DAGs were created (i.e., one with each expert). Important to note here is that the experts were not instructed to specify arrows between the newly-added variables. Figure 3 lists the identified time-varying variables and the number of experts that mentioned these variables.

Figure 3 shows that a large number of time-varying variables were identified by the experts. Considering that DAGs are necessarily a simplification of reality, the most relevant variables were selected by the first author for the final DAG by considering the timing of the variables and conceptual similarity. For example, the effect of sleep on PA was considered to be mediated by more proximal variables such as affect, physical complaints, and stress, which were already included in the DAG. Supplementary file 4 provides an overview of in- and exclusion decisions made for each variable and provides a rationale for the decision to exclude or rename specific variables.

**Fig. 4:**
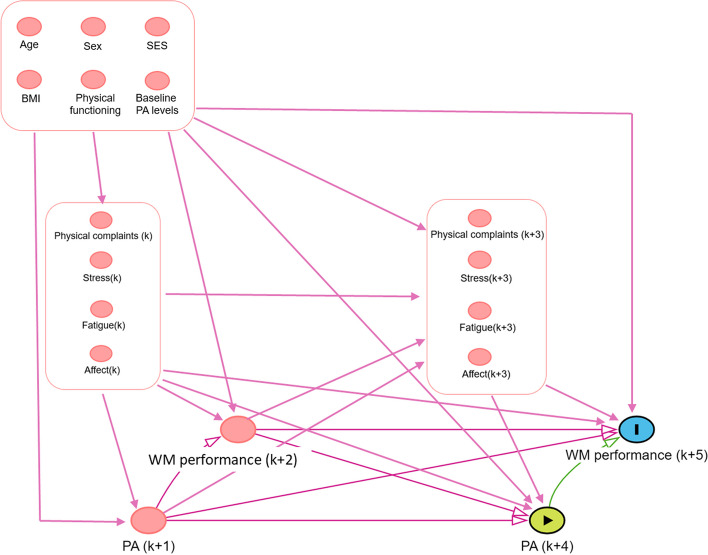
Developed DAG. Notes. SES = socio-economic status; BMI = body mass index; Rounded rectangles represent supernodes

Figure 4 shows the developed DAG. Two points are important to highlight here. First, to increase readability of the DAG, the time-invariant and the time-varying confounders were added into supernodes. The arrows leaving and entering a supernode are assumed to be relevant for all variables included in the supernode. The relations between variables in the same supernode are not specified. However, as all variables included in these supernodes influence both the exposure and the outcome, the specific relations between these variables are less relevant. Finally, the DAG was created by focusing on the cognitive domain EF instead of the subdomain WM. However, we do believe that the variables considered relevant for the cognitive domain are also relevant for its subdomains. The DAG clearly shows that an association between our exposure and our outcome can be generated by a causal relationship, but also by many unblocked backdoor paths (i.e., confounding bias). To distill the causal effect, efforts will have to be made to control the confounding.

## Results

There are two broad strategies for controlling confounding, namely physical control and analytic control [14]. The first strategy implies manipulation. As a result, the study design becomes (quasi-)experimental. The second strategy implies adjusting for confounding variables in the analysis phase. For observational studies, this is the only strategy for controlling confounding. Below we discuss both approaches in the context of an ESM study design.

### Physical control of confounding

In a randomized controlled trial (RCT) the impact of unblocked backdoor paths between the exposure and the outcome is eliminated by putting the exposure under experimental control. Doing so erases the arrows from the confounders to the exposure. This approach is, however, less feasible when we are interested in the immediate and short-lived effect of behaviors taking place in naturalistic environments rather than a lab setting.

**Fig. 5:**
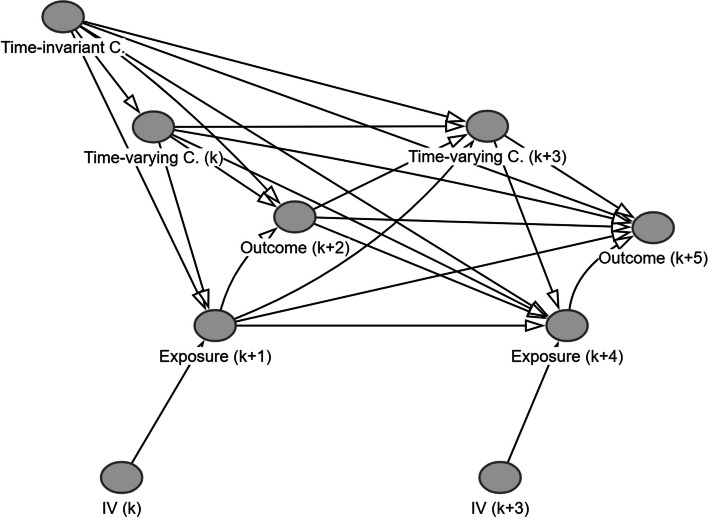
DAG visualizing the instrumental variable approach. Notes. IV = instrumental variable; C. = confounders; DAG created using Dagitty [32]

An alternative is the within-person encouragement design, introduced by Schmiedek & Neubauer [39]. In this design, participants receive encouragements to display the exposure of interest (e.g., performing PA at a moderate to vigorous intensity for a period of 15 min) across occasions on random timepoints (e.g., an encouragement is provided on day 1 and 4, but not on day 2 and 3). These encouragements then act as a time-varying *instrumental variable*. An instrumental variable is a variable that causes the exposure and is unrelated to the outcome other than through the exposure. In other words, the effect of the instrumental variable on the outcome is fully mediated by the exposure. Because potential confounders have no impact on the time-varying instrumental variable (since the encouragements are randomly provided), confounding bias is eliminated. Figure 5 shows the DAG visualizing this approach. We see that the encouragements (i.e., the time-varying instrumental variable) only influence the exposure. As they are provided at random timepoints, no arrow is drawn from the confounding variables to the encouragements. Arrows are still drawn from confounding variables to the exposure. This is because the exposure is not completely under experimental control. Participants are encouraged to display the behavior, but other aspects (e.g., fatigue or pain) might still make the participant decide to ignore the encouragement for PA. However, by adopting this design, researchers are able to filter out the variation in the exposure that is created by the encouragements and is thus under experimental control [16].

#### Illustrative example for physical control of confounding

**Fig. 6:**
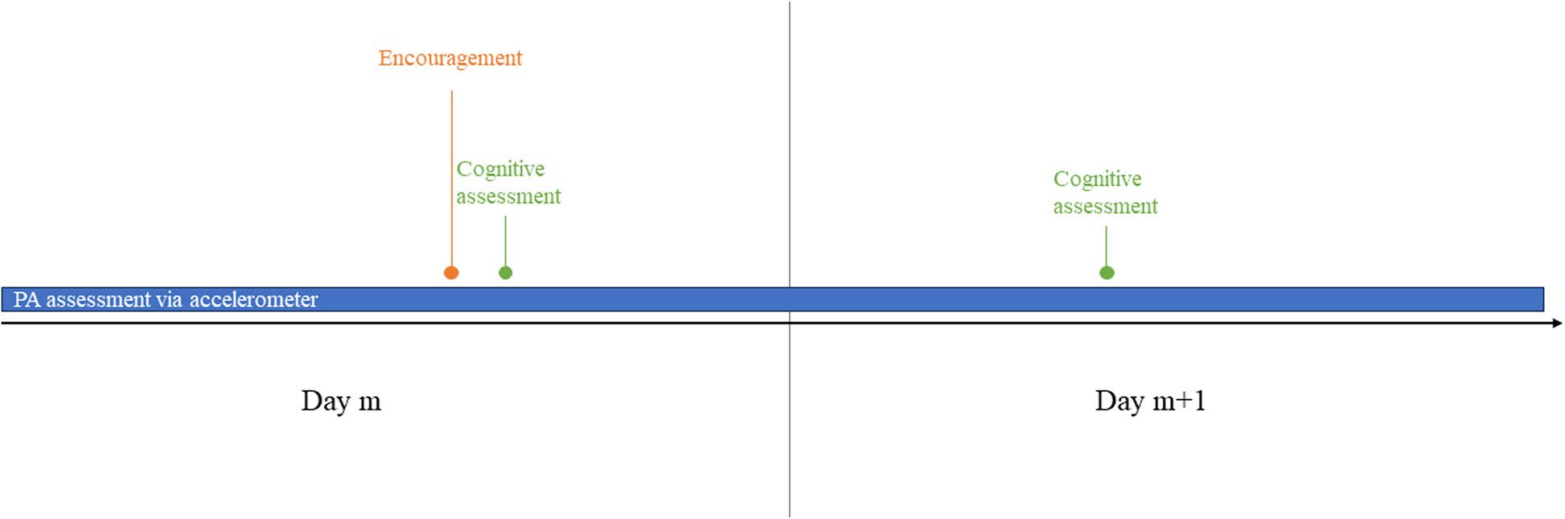
Design using a time-varying instrumental variable

For our use case, participants receive encouragements to perform PA at a moderate-to-high intensity for a period of ≥ 11 consecutive minutes before completing the cognitive assessment on randomly selected days during the study period. The percentage of days on which an encouragement to be physically active is provided and the timing of these encouragements should be discussed with the participants and can be individually adapted [39]. After receiving the encouragement, participants have a specific time period (e.g., 3 h) to display the behavior and to complete the cognitive assessment as soon as they have completed the behavior. The lengths of this time period can be decided based on a pilot study examining the feasibility to comply with the encouragement within different time periods. If participants do not respond to the prompt, the cognitive assessment is provided after the selected time period (e.g., 3 h). On non-encouragement days, participant receive a notification to complete the cognitive assessment on a random moment during the day (e.g., between 9 AM and 9 PM). Figure 6 visualizes the design for the instrumental variable approach.

### Analytic control of confounding

**Fig. 7:**
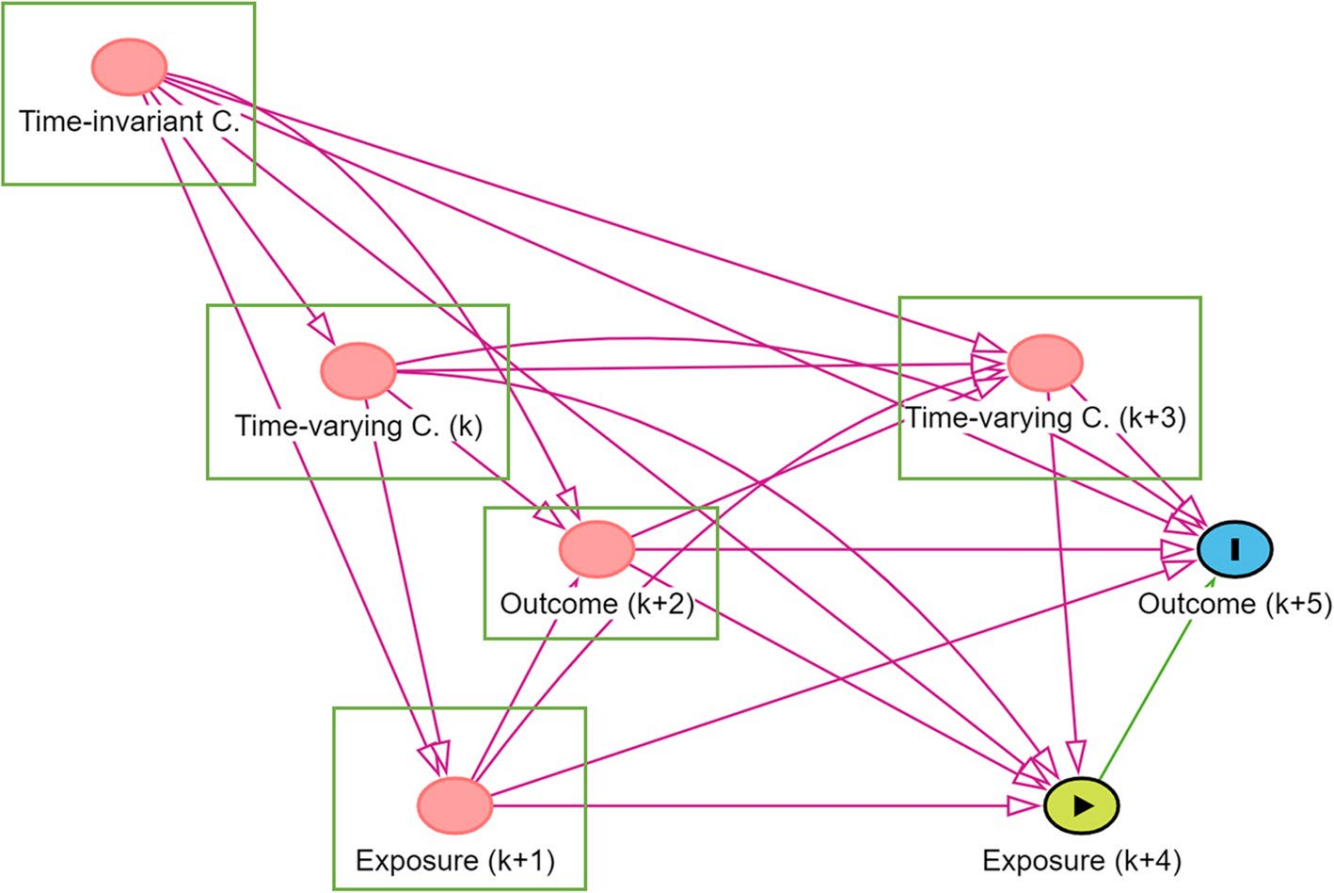
DAG visualizing analytic control of confounding. Notes. The green rectangles indicate the variables that need to be adjusted for in the analysis (i.e., the adjustment set); C. = Confounders

Another approach is adjusting for the confounding variables in the data-analysis phase [14]. Figure 7 displays this approach. To examine the causal effect of the exposure (k + 4) on the outcome (k + 5), we need to close all backdoor paths. Hence, we need to adjust for the time-invariant confounders, the time-varying confounders on timepoints k and k + 3, the exposure on the previous day (k + 1) and the outcome on the previous day (k + 2). This approach requires that, besides the exposure and outcome, all the confounding variables are assessed in the study. To take into account the correct temporal ordering of confounders, exposure and outcome, special attention should be paid to the timing of the assessments. The time-varying confounders should be assessed before the exposure and the exposure should be assessed before the outcome.

#### Illustrative example for analytic control of confounding

**Fig. 8:**
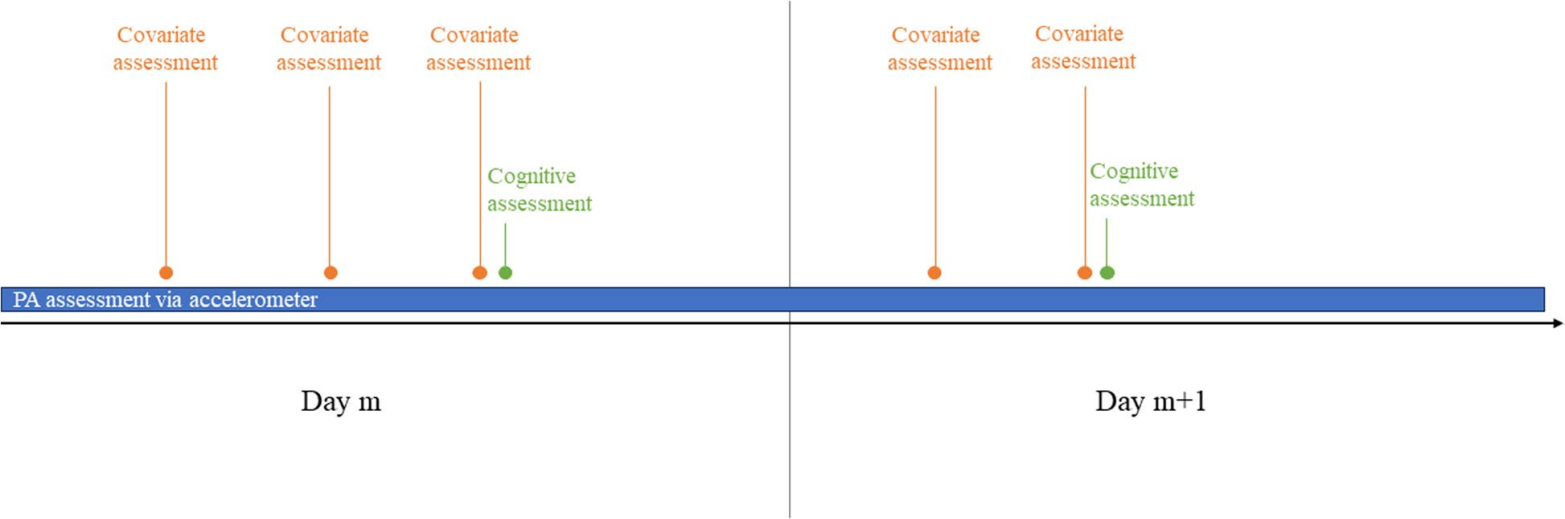
Design for analytic control of confounding

At the start of the study participants complete a questionnaire assessing the time-invariant confounders. Then, for the duration of the study, participants complete the cognitive assessments each day on a random moment between 10 AM and 6 PM. The decision to not assess EF in the evening is based on the finding that Flemish older adults’ activity levels tend to be very low in the evening [40]. The number of consecutive minutes of moderate-to-high intensity PA performed in the 25 min before the start of the cognitive assessment is extracted and categorized as ≥ 11 min or 0 min. Ideally, the time-varying confounders are assessed as close as possible to the start of 25-min interval before the cognitive assessment. For example, participants are prompted to report their physical complaints, affect, stress, and fatigue one hour before the start of the 25-min interval preceding the cognitive assessments. A reminder can be sent after 10 min in case participants did not respond to this prompt. However, to ensure that the assessment of the time-varying confounders takes place before the exposure, the questionnaire will be closed 60 min after the first prompt. Consequently, the earliest notification to complete the questionnaire can be sent at 8:35 AM. As one might expect reactivity to this schedule (i.e., people might show less PA because they are expecting the cognitive assessment shortly after the covariate assessment), one can assess the time-varying confounders multiple times before the cognitive assessment. A pilot study is recommended to evaluate whether including repeated assessments of the time-varying confounders can mitigate these potential reactivity effects. Figure 8 visualizes this approach for our use case.

In the analysis phase, we will adjust for the variables creating a spurious association between the exposure and the outcome. Based on Fig. 4, we can determine the minimal set of variables that we should adjust for. These are the time-invariant confounders (i.e., age, sex, socio-economic status, body mass index, physical functioning, and baseline PA levels), the exposure assessed on the previous day, the outcome assessed on the previous day, and the time-varying confounders assessed on the previous day and the same day (i.e., physical complaints, stress, fatigue, and affect).

### Summary

The developed DAG shows multiple backdoor paths creating confounding bias, even with temporal separation of exposure and outcome. We discussed two approaches for addressing this bias in ESM research, namely physical control and analytic control. Table 1 summarizes both approaches.

**Table 1:** Summary of physical control and analytic control approaches

	**Physical control approach**	**Analytic control approach**
Description	Exposure is manipulated to reduce or eliminate its association with confounding variables	Confounding variables identified by the DAG are assessed and statistically adjusted for during the estimation phase
Design type	(Quasi-)Experimental	Observational
Advantages	• Eliminates need to identify all confounders • Likely to create more variation in levels of the exposure	• Does not require a manipulable exposure
Limitations	• Requires a manipulable exposure • Participants must be willing to place the behavior of interest under experimental control	• Relies on accurate identification and assessment of all confounders • Requires precise timing of the assessment of exposure, outcome, and covariates

## Discussion

Due to technological advancements, studies using ESM offer exciting opportunities. We are now able to assess a broad range of people’s thoughts, emotions, experiences and actions in real time and in naturalistic settings. Furthermore, we can augment these data with real-time information on people’s environment (e.g., location, weather, and noise). These advancements create avenues for further research. However, the innovativeness of ESM studies might have, as Rohrer and Murayama nicely describe it, “led people to put the technological and methodological cart before the conceptual horse” [9] (page 7). This paper demonstrates the relevance of the identification phase for ESM studies aiming to answer causal research questions.

Traditionally, the following steps have been proposed for developing an ESM study: (1) define the research question, (2) determine the study design, (3) develop questionnaires suitable for repeated assessments, (4) select a platform for scheduling and delivering remote assessments, and (5) perform the analysis [45]. For causal research questions, it is essential to adopt an explicit causal approach and clearly articulate the causal nature of the research question in step 1. This approach requires adding an additional step: the identification phase. By clearly defining the causal question and the underlying assumptions during the identification phase, researchers obtain critical information to inform the subsequent steps, particularly the study design and the analysis. This approach is likely to enhance the study’s internal validity, thereby yielding more accurate and actionable insights to inform intervention development.

Some have argued that, due to their ability to temporally separate the exposure from the outcome, observational ESM studies can provide information on ‘potential causal sequences’ [7]. Temporality is necessary for inferring causation, but not sufficient [41, 42]. By developing a DAG for our estimand we demonstrated that there were many open backdoor paths linking our exposure and outcome, even when the exposure is assessed immediately before the outcome. To identify these backdoor paths, literature as well as experts were consulted. Interestingly, none of the identified papers examining the effect of PA in a naturalistic environment on EF at day-level included time-varying confounders, highlighting the relevance of outlining the assumed causal structure between the exposure and the outcome using a DAG.

The impact of open backdoor paths (i.e., confounding bias) can be reduced using physical as well as analytic control [14]. When physical control is possible (i.e., the exposure can be manipulated); the decision to perform an RCT or a study using a within-person encouragement design depends upon the estimand. If we would be solely interested in the effect of PA on WM performance, we should perform an RCT. However, in our case, we were interested in the effect of PA performed in a naturalistic environment rather than in a lab setting. Furthermore, from a health promotion perspective, an encouragement to perform the behavior is also more closely related to an intervention promoting PA than a setting in which one is forced to perform the behavior. In this way, the within-person encouragement design also differs from other within-person experiments, such as for example ABAB designs [43].

In comparison with a purely observational ESM study design, the within-person encouragement design offers a number of advantages. The most important advantage is that this approach does not require researchers to assess and adjust for all the confounding variables identified in the DAG. A second important advantage of this design is that it is likely to create more variation in the levels of the exposure than we would obtain in a purely observational study. A study among our target group of older adults showed that the median number of minutes of PA at moderate to high intensity per day was 10 (range: 3 – 24) [40]. Hence, in a purely observational study, the number of occasions on which older adults would perform the cognitive assessments after performing ≥ 11 min of PA at a moderate to high intensity might be very limited. Rohrer and Murayama also argue that limited within-person variability in the exposure might be an underlying reason for the differences found between within- and between-person effects [9]. Hence, for ESM studies examining the causal effect of behaviors that are under participants’ control and can be repeatedly observed in daily life (e.g., PA, breakfast consumption, or performing breathing exercises) [39], the within-person encouragement design offers an interesting alternative to address confounding bias.

It is important to note that the within-person encouragement design provides different information than the observational longitudinal design. Using the observational design, we will obtain the *average treatment effect* or the causal effect of the exposure on the outcome across occasions and participants. In contrast, using the within-person encouragement design we obtain the *local average treatment effect (LATE)* or the causal effect of the treatment for occasions on which the participants acted in accordance with the prompt [39]. For our case, this would be the causal effect for occasions on which participants actually performed ≥ 11 min of PA at a moderate to high intensity in their natural environment when they received the encouragement and did not do so when they did not receive the encouragement. However, considering the rise in just-in-time adaptive interventions aiming to provide users with prompts optimized to situations during which they are able and willing to show the targeted behavior [44], the LATE might exactly be the effect that we are interested in.

In many cases, one is interested in exposures that are not controllable due to practical or ethical reasons (e.g., stress levels or self-efficacy to be physically active). For this type of research questions, analytic control will be the only option to reduce confounding. As demonstrated above, the timing of the assessment of the covariates is key in order to distill the causal effect of the exposure on the outcome. In contrast with studies using retrospective assessments, a key strength of ESM studies is that researchers are indeed able to specify the timing of the assessment of specific variables throughout the day. However, as questionnaire density negatively impacts data quality and compliance [45], the number of covariates that can be assessed per measurement occasion will be limited. Again, this highlights the relevance of creating a DAG to select the most relevant covariates for assessment in the study.

As this paper demonstrates, the identification phase can be time-intensive and demands domain expertise. Additionally, developing a DAG capturing complex temporal dynamics is challenging. Moreover, it may be infeasible to control for all potential sources of confounding. Importantly, bypassing this phase does not eliminate the need for assumptions. Instead, it leaves the causal question and the associated assumptions vague and underspecified, possibly leading to biased estimates, ambiguous interpretations and unwarranted conclusions [17]. At the very least, creating DAGs adds to the transparency of the assumptions made or the potential sources of bias that could not be adjusted for.

A first limitation of this paper is that we only focused on confounding bias. A spurious association between the exposure and the outcome can, however, also be created by other types of biases, such as collider and measurement bias [16]. For example, in the context of ESM studies, collider bias occurs when participants’ likelihood to respond to the prompts is influenced by their exposure and outcome values (e.g., low levels of PA and feeling less cognitively sharp). Measurement bias can have different structures [16]. For example, it could be the case that on days on which participants have suboptimal WM performance, they are more likely to wear the accelerometer incorrectly. As a result, a spurious path is created between the exposure and the outcome. Readers interested in collider and measurement bias are referred to Chapters 8 and 9 of Hernán and Robins [16]. Second, in the current paper, we provide limited guidance for the estimation or data-analysis phase. For both illustrated approaches, different parametric models can be used. For example, estimating causal effects using instrumental variables can be done via multiple regression as well as structural equation modeling [46]. Similarly, the selection of a parametric model for analytic control of confounding (e.g., cross-lagged panel model), will depend on a number of parametric assumptions and the presence of unmeasured confounding variables [47]. By refraining from describing a parametric model for both approaches we aim to mitigate creating the incorrect impression that each approach is linked with a specific estimation technique. Furthermore, as indicated in the Introduction section, we aimed to highlight the relevance of the identification phase as a distinct and independent step in causal inference.

## Conclusions

This paper demonstrates the usefulness of the identification phase for ESM studies aiming to answer causal research questions. We highlighted the need for more clarity and transparency regarding the causal research question and showed how researchers can effectively communicate their causal question and its underlying assumptions using DAGs. Furthermore, we illustrated both the physical and analytic control approach using an illustrative example in the context of ESM study design in the domain of PA. For research questions in which the exposure is manipulable (e.g., PA and diet), we believe that the within-person encouragement design offers interesting avenues for further ESM research.

## Supplementary Information

Supplementary Material 1.

Supplementary Material 2.

Supplementary Material 3.

Supplementary Material 4.

## Data Availability

Data sharing is not applicable to this article as no datasets were generated or analysed during the current study.

## Abbreviations

ESM: Experience sampling method
PA: Physical activity
DAG: Directed acyclic graph
EF: Executive functioning
WM: Working memory
RCT: Randomized controlled trial
LATE: Local average treatment effect
