# Supplementary material for: Experience sampling method studies in physical activity research: the relevance of causal reasoning
Source: Int J Behav Nutr Phys Act. 2025 Mar 5;22:28. doi: 10.1186/s12966-025-01723-w (PMC11884166; doi:10.1186/s12966-025-01723-w)
Supplement: Supplementary file 1 — Supplementary Material 1. [file 12966_2025_1723_MOESM1_ESM.docx]

Evidence synthesis based on the literature

First, the conclusions of each study were mapped into an ‘implied graph’ (*mapping phase*). Hence, for each study, the outcome and the exposure were added to the graph and all control variables were added as unassigned variables. Then, directed edges were drawn from the control variables to the outcome and the exposure. Furthermore, edges between each of the control variables were added. The direction of the edges between the control variables did not matter at this phase as they would be re-examined in the following phase [1]. Consequently, the end-product of the first phase was a saturated DAG (i.e., a DAG in which all variables are connected with each other) for each of the studies. Figure 1 shows the implied graph for the study of Phillips et al. [2].


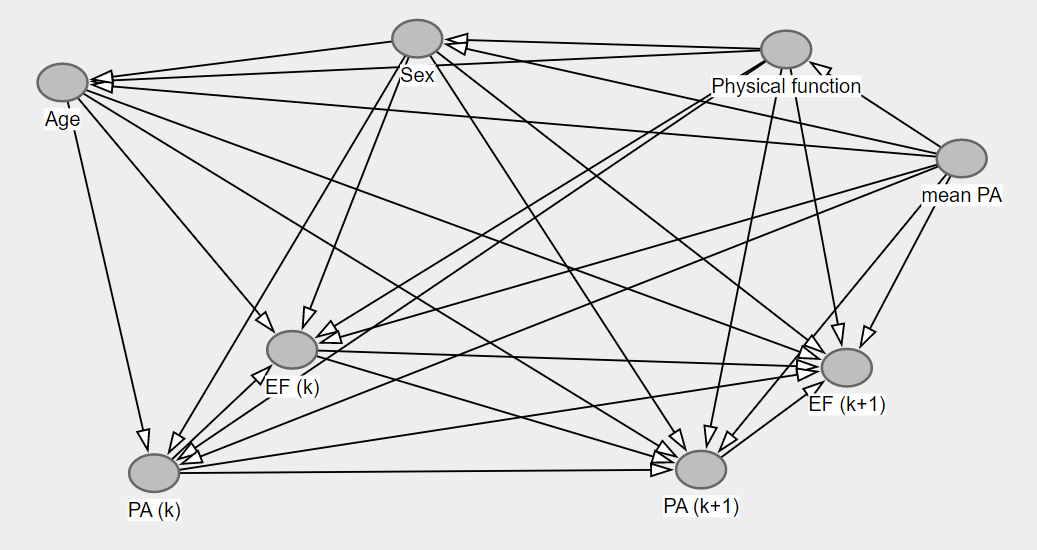


Figure 1. Implied graph for the study of Phillips et al. [2].

The second phase involves applying causal theory to each relation in the implied graphs (*translation phase*) [1]. In this phase, each edge in the implied graph is assessed based on four criteria. A first criterium is temporality: an effect cannot take place before the cause. Plausibility is a second criterium. Based on the researchers’ background knowledge implausible relations can be reversed or removed. Ferguson et al. describe this criterium as “face-validity” [1]. However, because face-validity is often considered a misnomer [3], we named this criterium “plausibility”. A third criterium is recourse to theory. In this step formal theoretical support for the relation is assessed. The final criterium is a counterfactual thought experiment. To assess this criterium, we compare counterfactual exposures and consider whether the potential outcomes would be different. For example, for the directed edge between age and PA at timepoint k (PA(k)) the counterfactual thought experiment would focus on whether we would expect the same average total volume of PA for older versus younger people. If we would expect different scores, the directed edge from “age” to “PA(k)” should be retained. If any of these causal criteria is not present, the edge can be removed [1]. However, because lack of theory does not necessarily imply a lack of effect, the criterium ‘recourse to theory’ should not be the sole reason for removing an edge [1]. This second phase resulted in a DAG for each study. Figure 2 shows the DAG for the study of Phillips et al. [2].


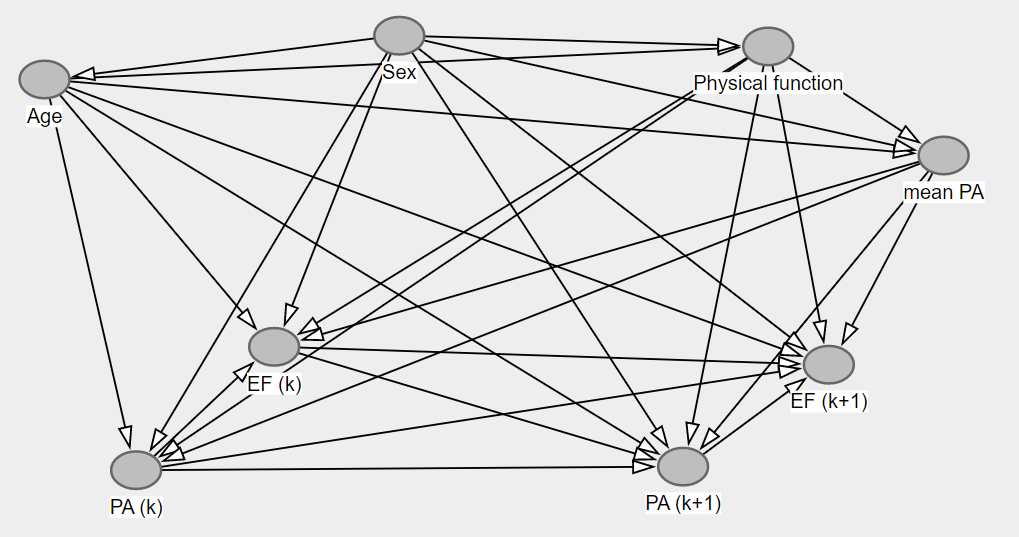


Figure 2. DAG for the study of Phillips et al. [2].

In the third phase the directed edges from each of the developed DAGs are integrated into the I-DAG *(integration phase)* [1]. To do so, each of the directed edges is transferred to a blank DAG. Figure 3 provides the result of this phase.


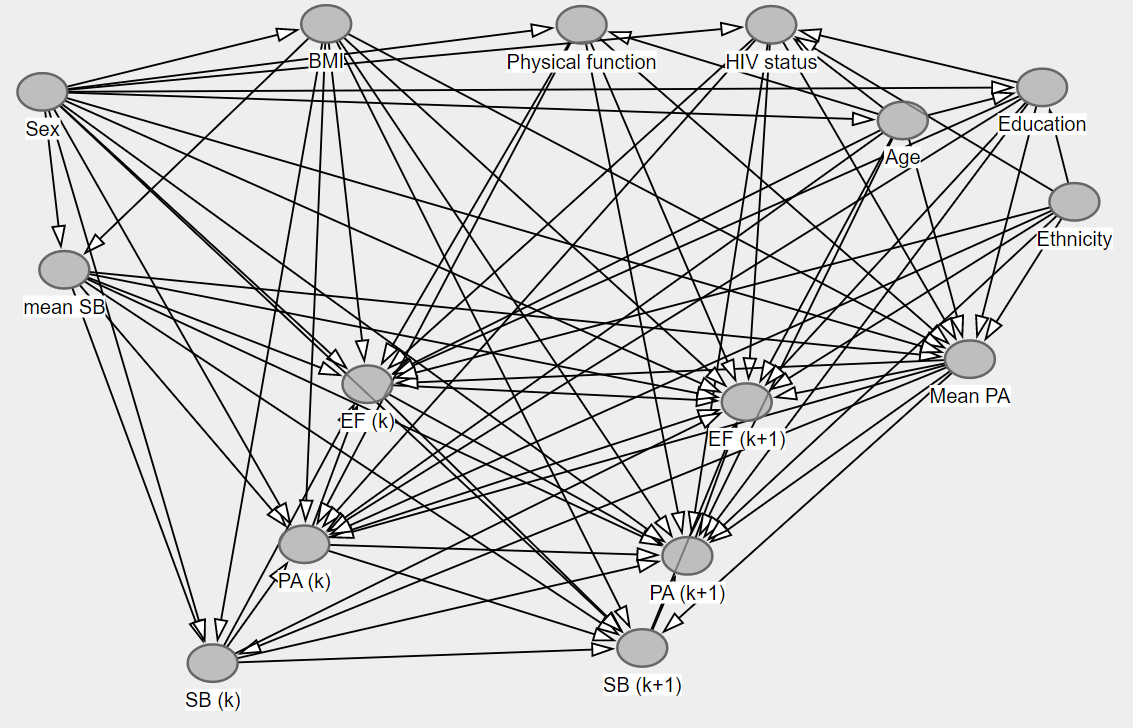
Figure 3. Integrated DAG.
Notes. SB = sedentary behavior. BMI = body mass index

Several actions were taken to reduce the complexity of this I-DAG. First, the nodes related to sedentary behavior were removed. These nodes were added based on the study of Fitzsimmons et al. in which sedentary behavior was also considered as a predictor of daily cognitive abilities [4]. In the current study, we will focus on the difference between obtaining 0 versus ≥11 minutes of PA at a moderate to vigorous intensity in the 25 minutes interval before the start of the cognitive assessments. Hence, it was not considered relevant to add sedentary behavior to the graph. Second, similar nodes were recombined into one node. Recombination of nodes is considered acceptable when there is theoretical support for doing so (e.g., the nodes refer to different facets of the same construct) and when they have identical in- and output [1]. Here it was decided to combine “physical functioning” and “HIV status” into one node called “physical functioning”. Furthermore, “education” and “ethnicity” were combined into “socio-economic status (SES)”. Finally, each of the three studies included ‘mean PA’. Adding the person-level mean of the exposure is an often used strategy to distill the within-subject effect from the between-subject effect and to reduce the impact of unmeasured cluster-level confounders [5, 6]. However, as the person-level mean combines information over the whole study period, it becomes difficult to define this variable as a common cause of the exposure and the outcome. Hence, it was decided to include ‘baseline PA’ (i.e., level of PA before the start of the study) as common cause instead ‘mean PA’. After performing these steps, we checked whether new edges were possible. These were assessed using the steps described in the translation phase [1]. Figure 4 shows the result of these steps.


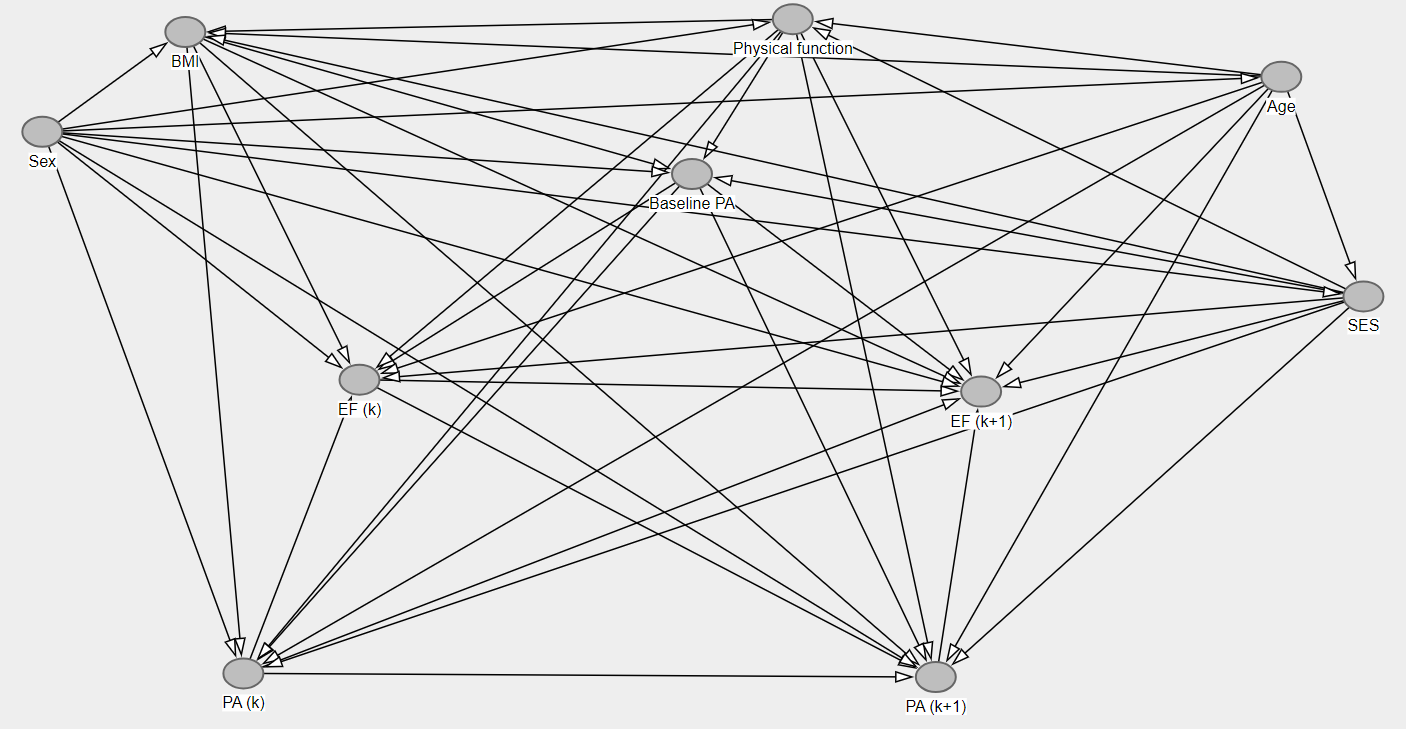


Figure 4. I-DAG after removing sedentary behavior and recombination of nodes.

**Bibliography**

1. Ferguson KD, McCann M, Katikireddi SV, Thomson H, Green MJ, Smith DJ, Lewsey JD: **Evidence synthesis for constructing directed acyclic graphs (ESC-DAGs): a novel and systematic method for building directed acyclic graphs.** *International journal of epidemiology* 2020, **49:**322-329.

2. Phillips CB, Edwards JD, Andel R, Kilpatrick M: **Daily physical activity and cognitive function variability in older adults.** *Journal of aging and physical activity* 2016, **24:**256-267.

3. Lilienfeld SO, Pydych AL, Lynn SJ, Latzman RD, Waldman ID: **50 differences that make a difference: a compendium of frequently confused term pairs in psychology.** In *Frontiers in Education*. Frontiers Media SA; 2017: 37.

4. Fitzsimmons PT, Maher JP, Doerksen SE, Elavsky S, Rebar AL, Conroy DE: **A daily process analysis of physical activity, sedentary behavior, and perceived cognitive abilities.** *Psychology of sport and exercise* 2014, **15:**498-504.

5. Neuhaus JM, Kalbfleisch JD: **Between-and within-cluster covariate effects in the analysis of clustered data.** *Biometrics* 1998**:**638-645.

6. Brumback BA, Li L, Cai Z: **On the use of between–within models to adjust for confounding due to unmeasured cluster-level covariates.** *Communications in Statistics-Simulation and Computation* 2017, **46:**3841-3854.
