# Supplementary material for: Experience sampling method studies in physical activity research: the relevance of causal reasoning
Source: Int J Behav Nutr Phys Act. 2025 Mar 5;22:28. doi: 10.1186/s12966-025-01723-w (PMC11884166; doi:10.1186/s12966-025-01723-w)
Supplement: Supplementary file 3 — Supplementary Material 3. [file 12966_2025_1723_MOESM3_ESM.pdf]

Directed acyclic graphs (DAGs)
short introduction

Expert consultation DAG development

Project aim

To examine the causal impact of physical activity on older adults' daily cognitive performance

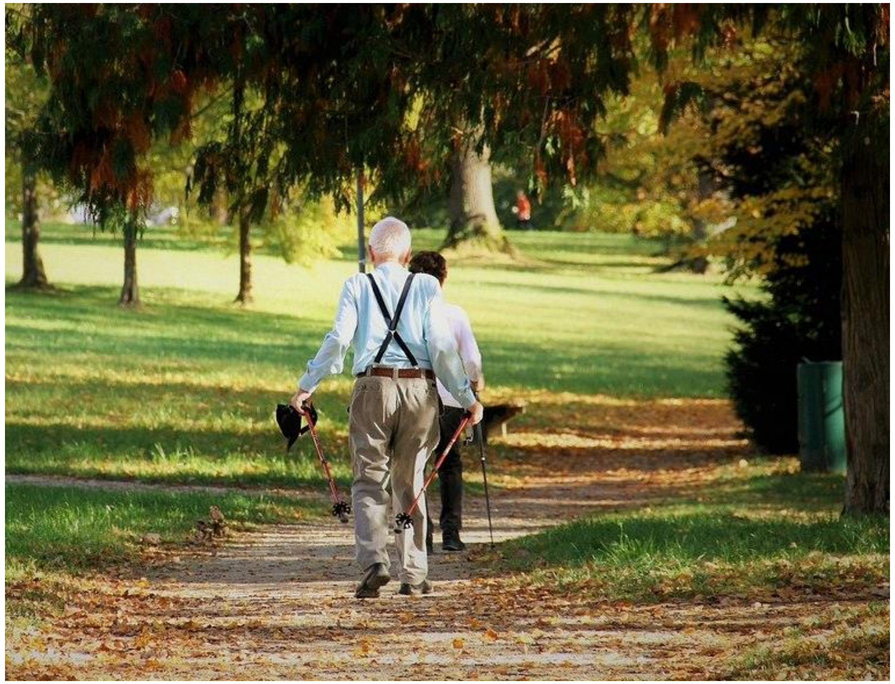

Causal effect or association?

Causal inference is a core task of health promotion research:

↳ Basis for developing guidelines and interventions to alter people's behaviour

However, when using observational designs, we often do not dare to say we aim to examine causal effects!

Causal effect or association?

We do not dare to speak about causation (with observational data)

↓

We do not think causally

↓

Our observed associations are probably biased

↓

We make (careful) causal conclusions

Interesting article on this topic: Hernan, AJPH, 2018, Vol 108 (5)

Introduction to DAGs: a paradox

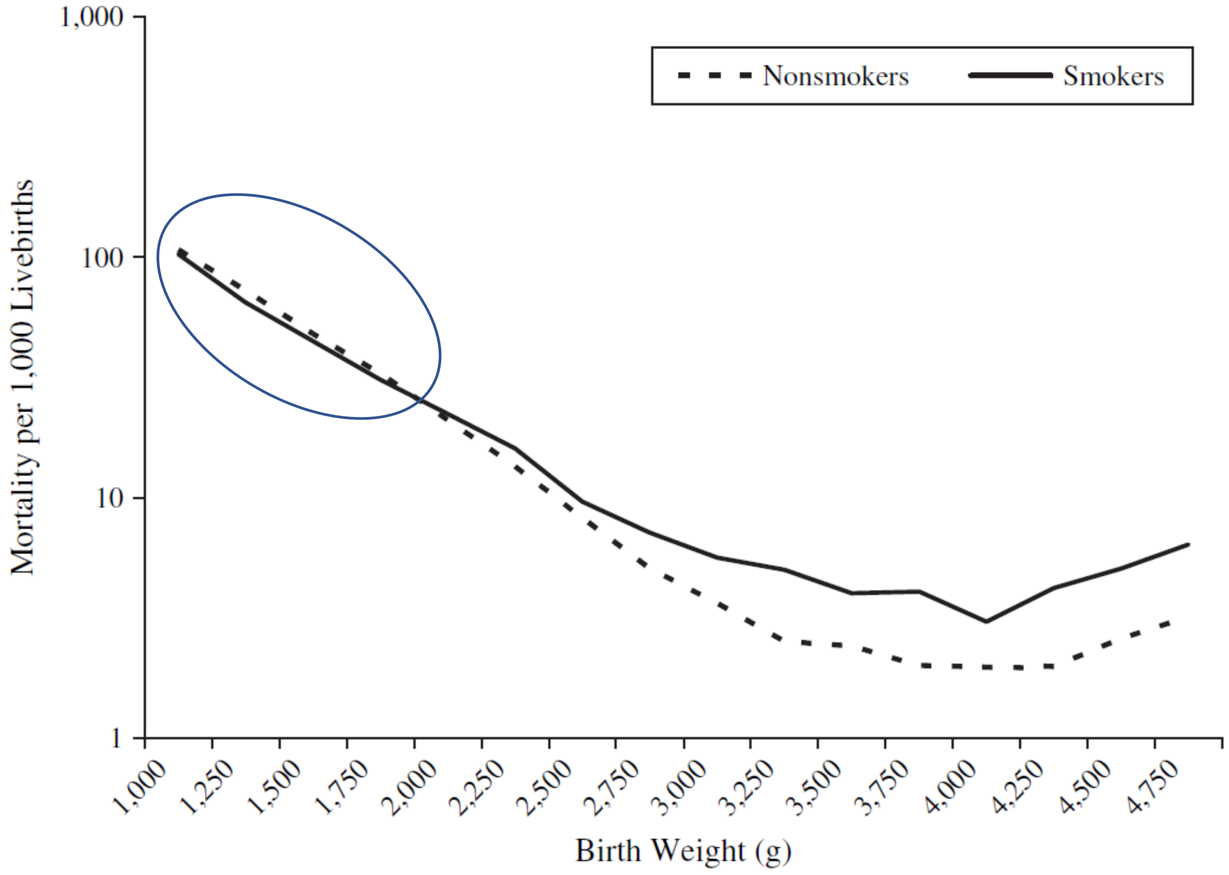

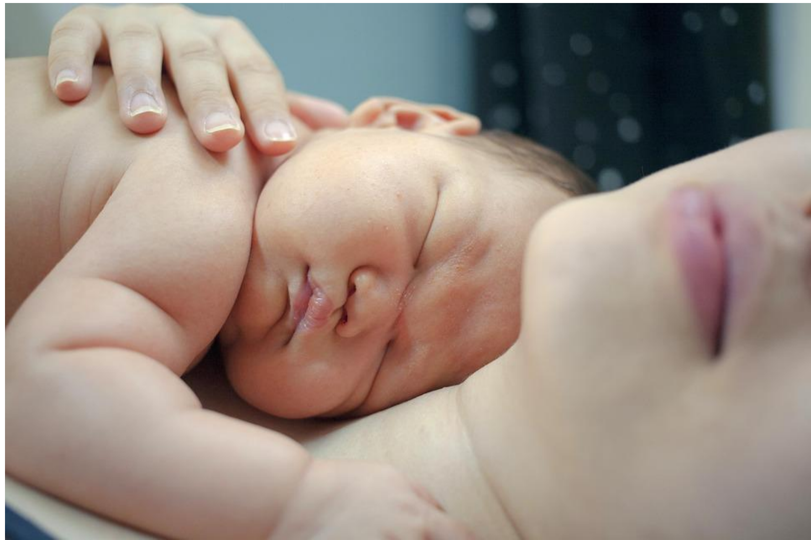

Five sources of association

1. Random fluctuation
2. 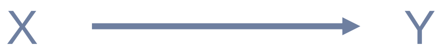

3. 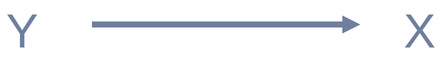

4. 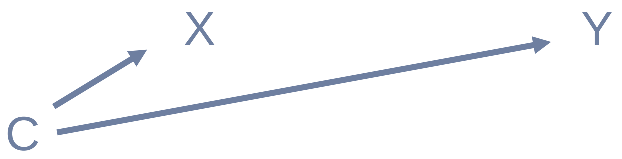

5. 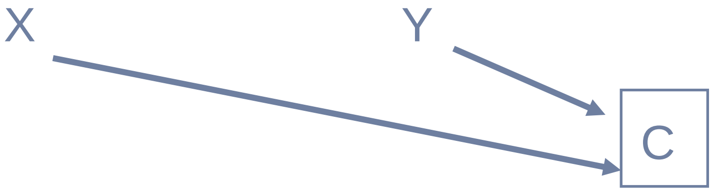

Source: Glymour, 2006

Five sources of association

1. Random fluctuation
2. PA → cognitive performance
3. Cognitive performance → PA
4. Age → PA; Age → cognitive performance
5. PA → Living independently; cognitive performance → Living independently

Directed acyclic graphs to the rescue!

What?

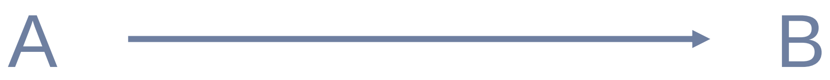

- Graphical system of nodes (variables) along with directed edges
- Visually encode researchers' a priori assumptions about causal relations among the exposure, outcomes and covariates
- Without cycles

Directed acyclic graphs to the rescue!

What?

How?

- Directed paths or causal paths
- Undirected paths or spurious paths

Directed acyclic graphs to the rescue!

What? | How?

Directed paths or causal paths

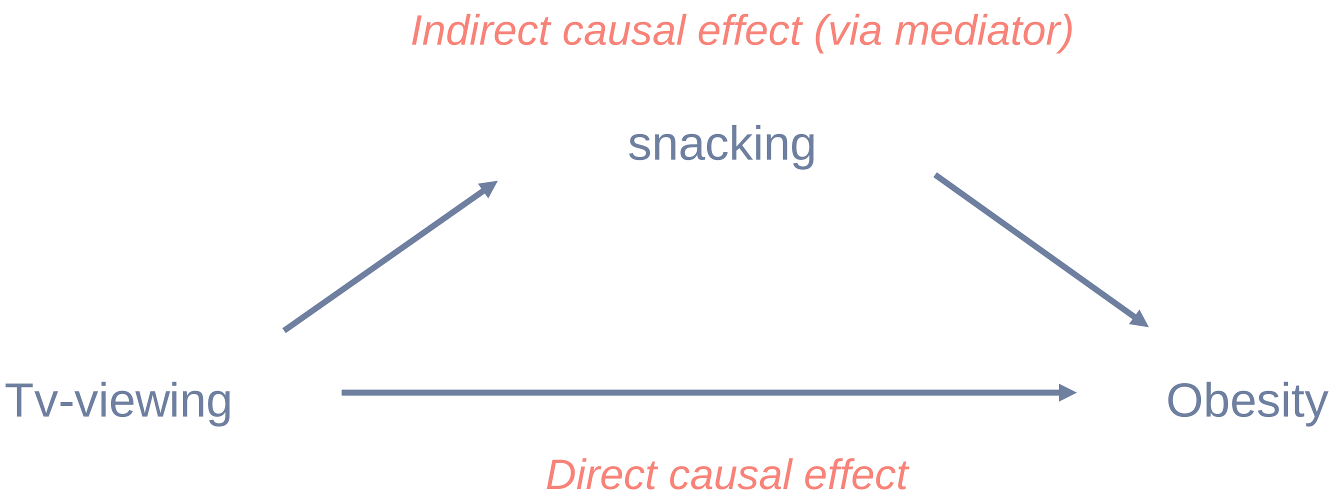

Directed acyclic graphs to the rescue!

What?

How?

Directed paths or causal paths

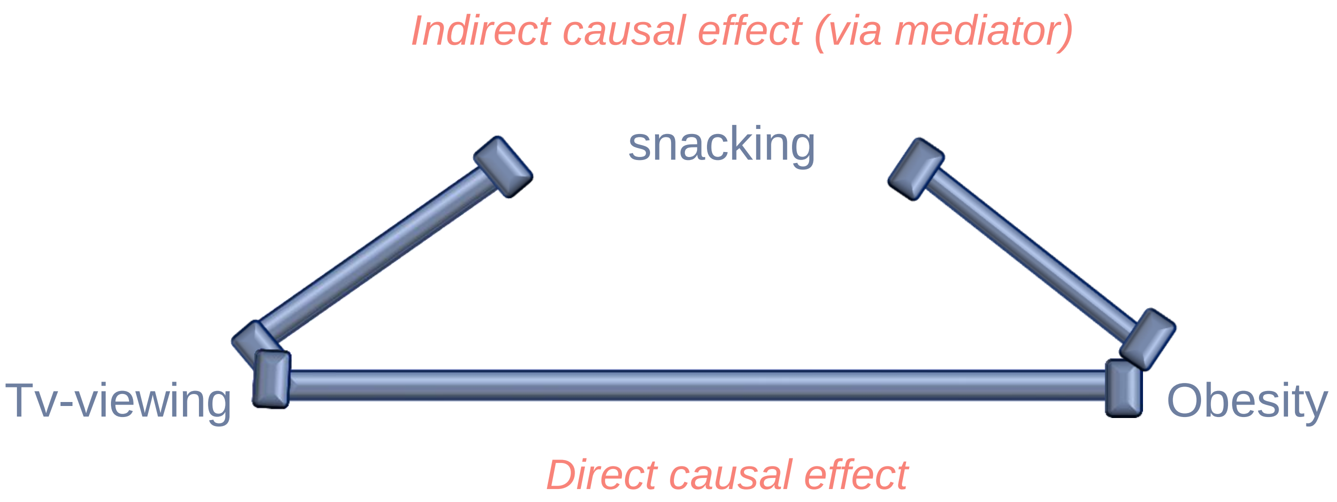

Directed acyclic graphs to the rescue!

What?

How?

Directed paths or causal paths

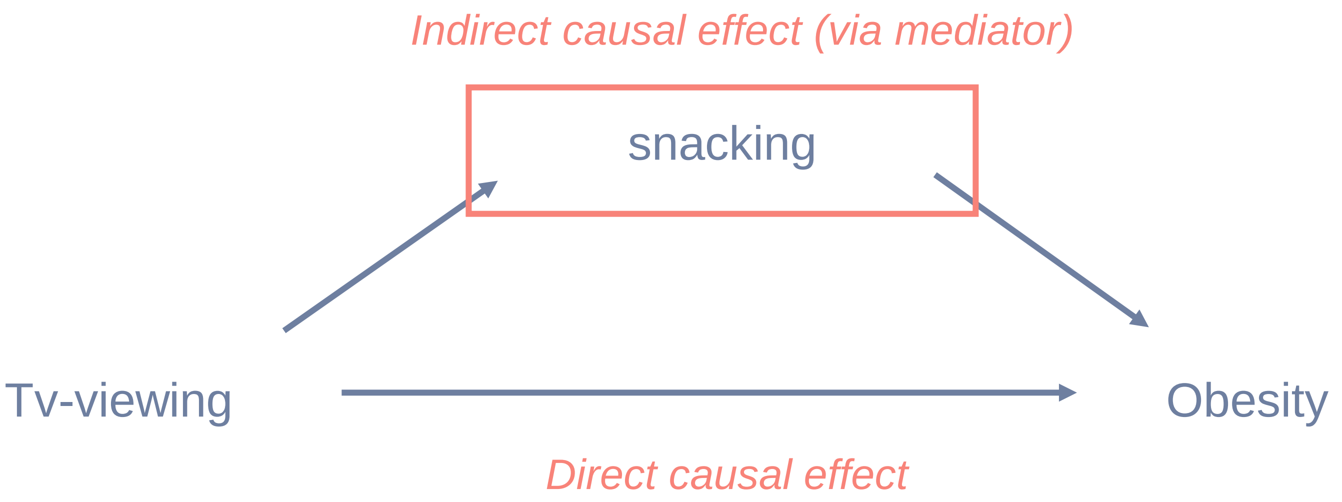

Directed acyclic graphs to the rescue!

What?

How?

Undirected paths or spurious paths

→ 2 variables share the same cause

→ Association transmitted by backdoor path is non-causal (confouding)

Confounding

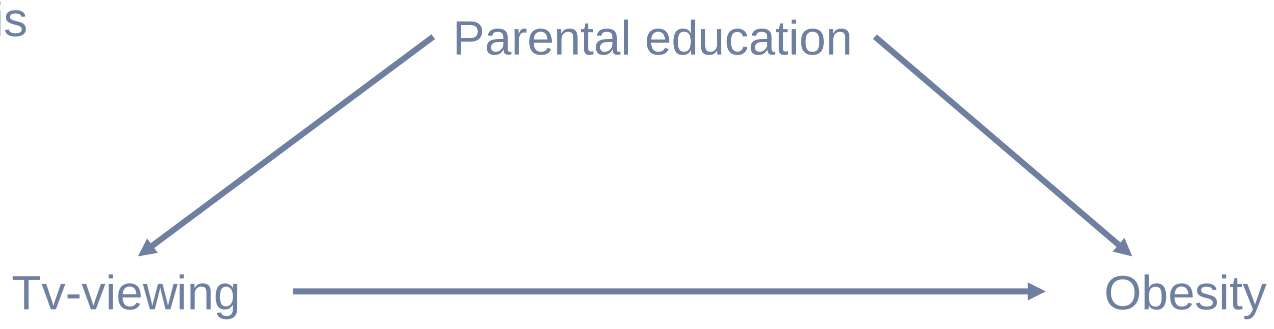

Directed acyclic graphs to the rescue!

What? How?

Undirected paths or spurious paths

→ 2 variables share the same cause

→ Association transmitted by backdoor path is non-causal (confouding)

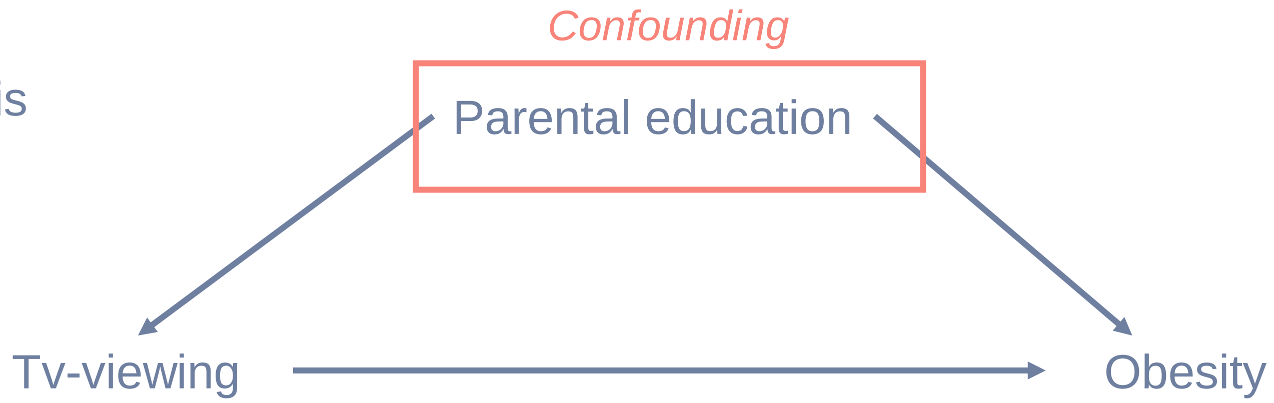

Directed acyclic graphs to the rescue!

What? How?

Colliders

→ 2 variables have the same effect

→ No associaton transmitted unless we control for it!

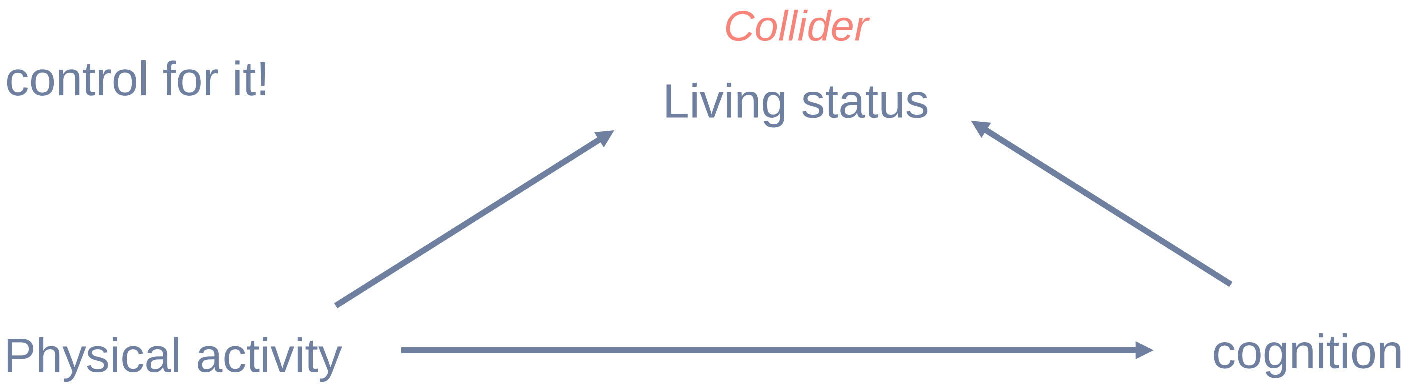

Directed acyclic graphs to the rescue!

What?

How?

Colliders

→ 2 variables have the same effect

→ No associaton transmitted unless we control for it!

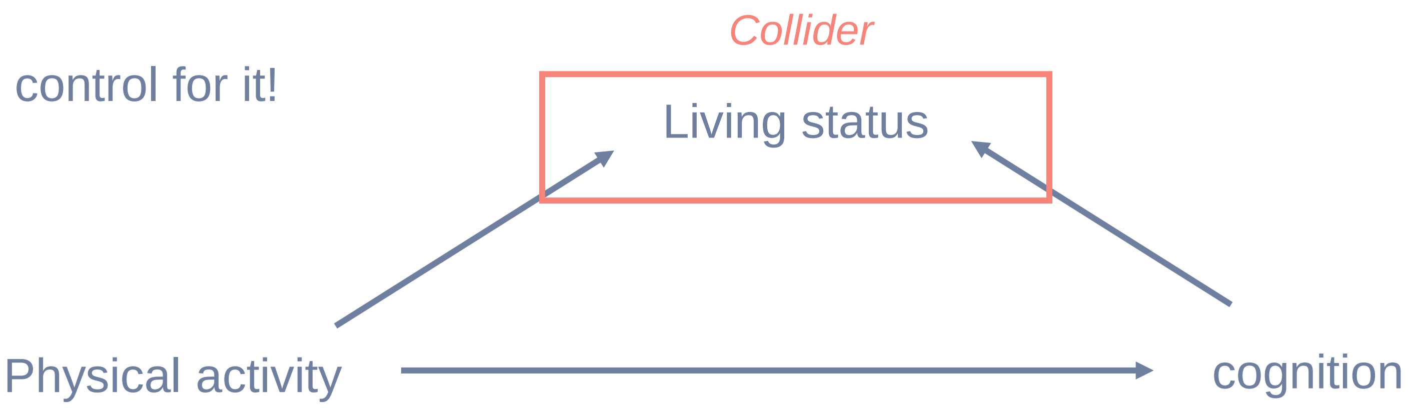

Directed acyclic graphs to the rescue!

What? | How?

D-separation

Change status of a path from open to close or vice versa by acting on a variable

- Via study design
- Via statistical adjustment

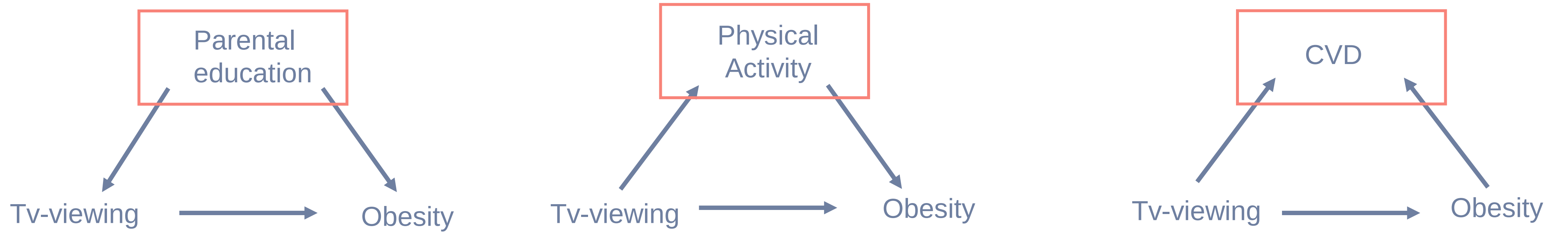

Directed acyclic graphs to the rescue!

What?

How?

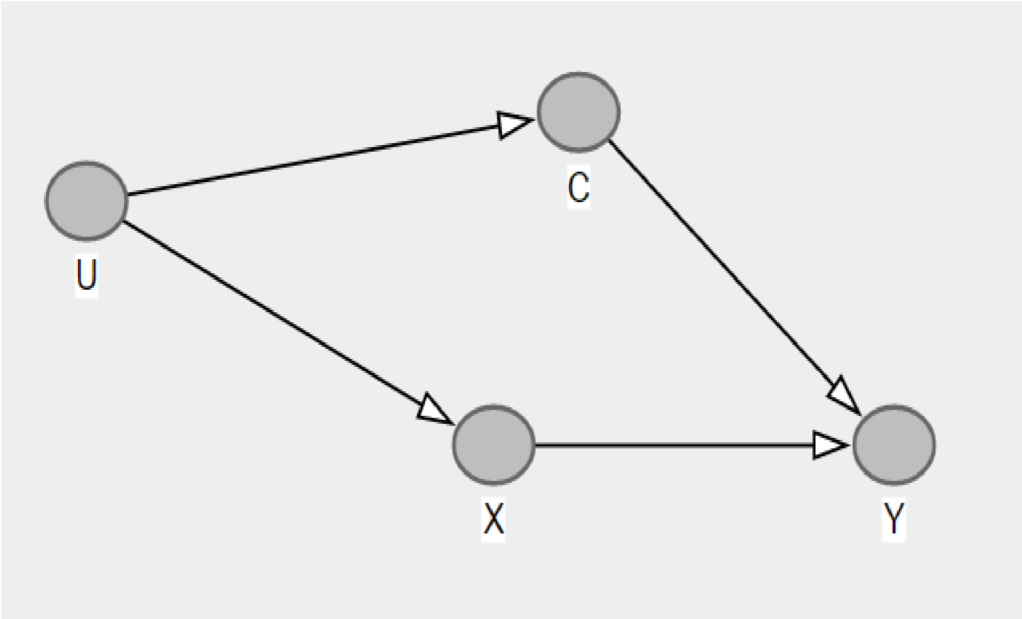

Want to estimate the causal effect of X on Y

C = measured variable

U = unmeasured variable

Directed acyclic graphs to the rescue!

What?

How?

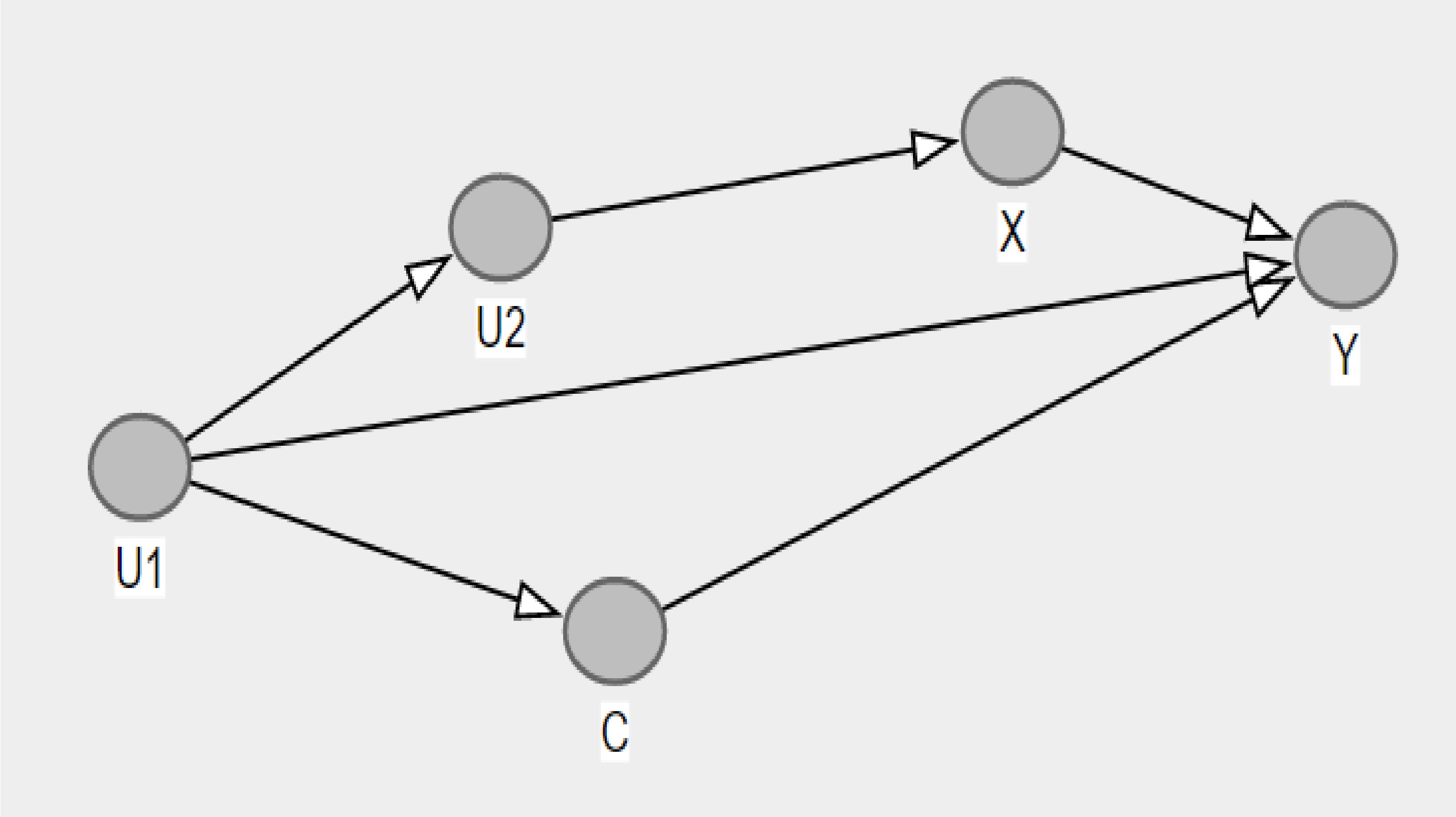

Want to estimate the causal effect of X on Y

C = measured variable

Us = unmeasured variables

Directed acyclic graphs to the rescue!

What? | How? | Assumptions

- No omitted confounding pathways
- Removing an arrow is a stronger assumption than adding an arrow

Directed acyclic graphs to the rescue!

What? | How? | Assumptions | Result

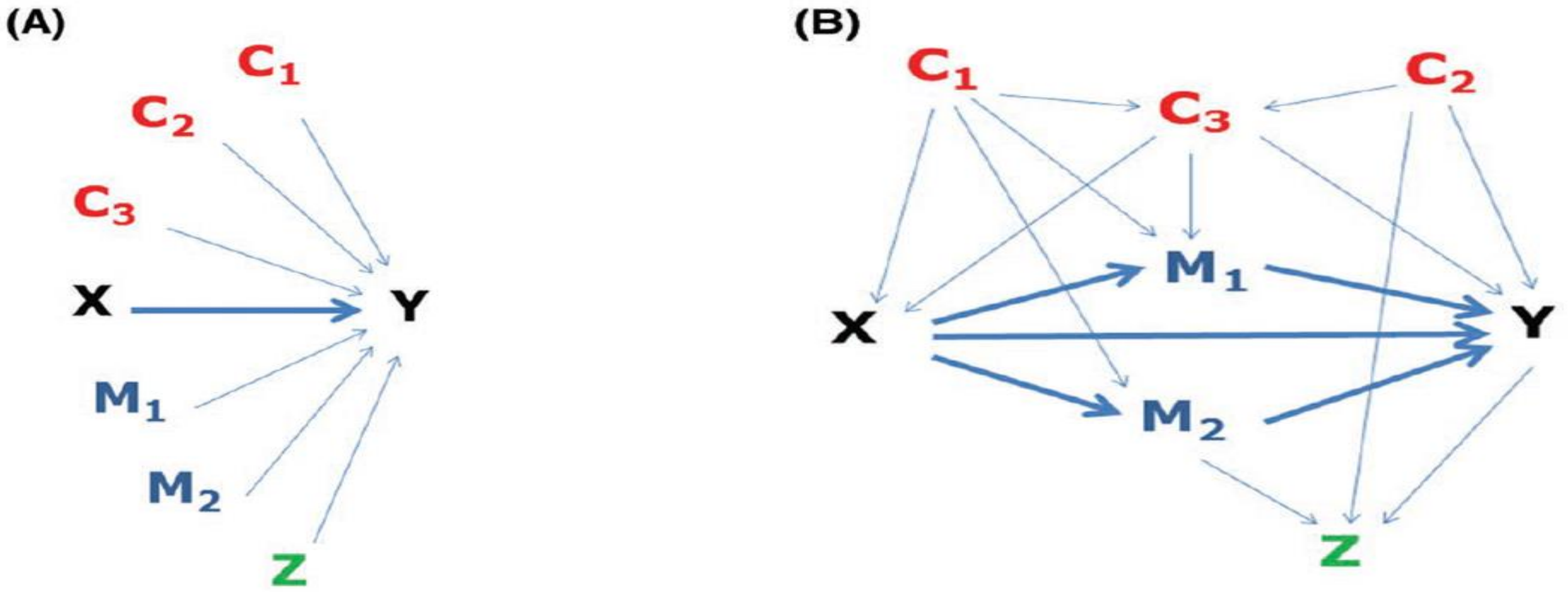

Source: Gaskell & Sleigh (2020)

Back to our paradox

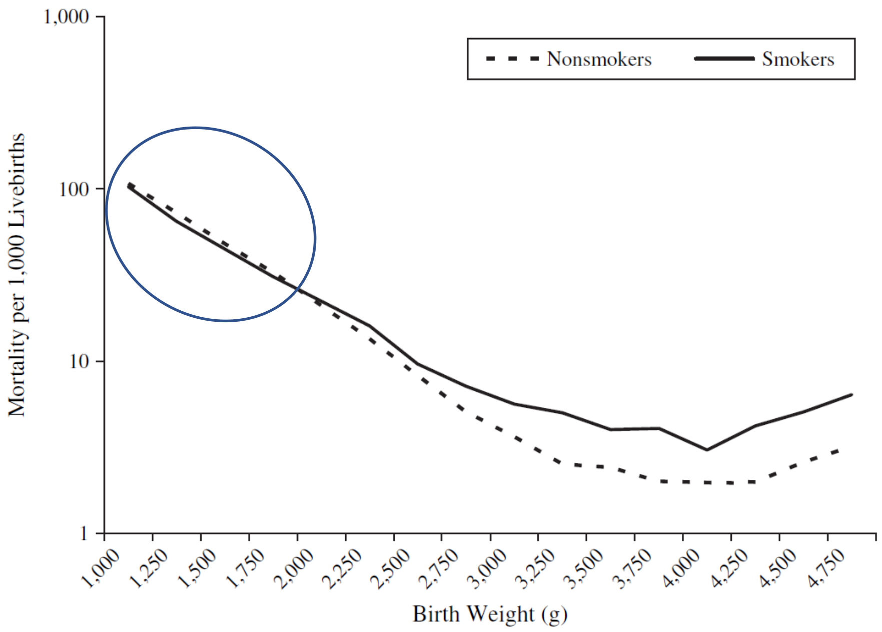

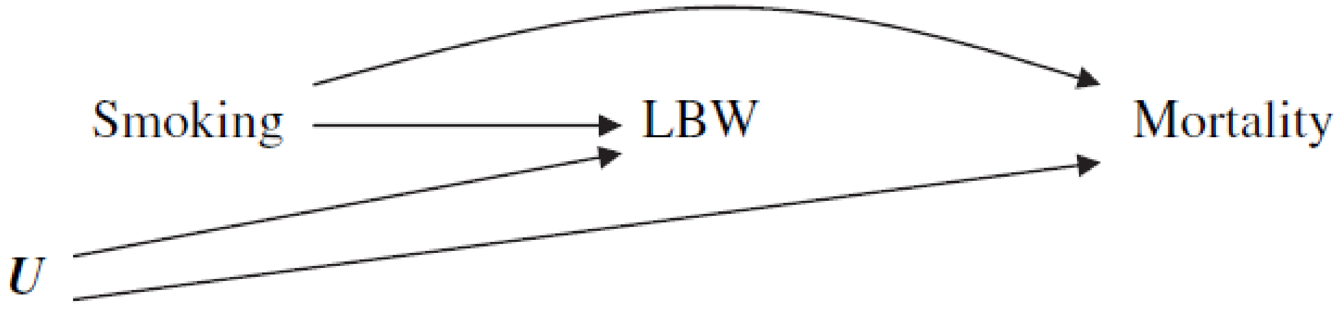

Source: Hernandez-Diaz et al. (2006)

Thanks

Louise Poppe

Expert consultation DAG development
